# Characterization of cognitive functioning in complex PTSD compared to non-complex PTSD

**DOI:** 10.3389/fpsyt.2024.1433614

**Published:** 2025-01-15

**Authors:** Zoe-Sofia Schöndorf, Valentin Terhoeven, Anna Jaehn, Daniela Roesch-Ely, Hans-Christoph Friederich, Christoph Nikendei, David Kindermann

**Affiliations:** ^1^ Department of General Internal Medicine and Psychosomatics, University Hospital Heidelberg, University of Heidelberg, Heidelberg, Germany; ^2^ DZPG (German Centre for Mental Health – Partner Site Heidelberg/Mannheim/Ulm), Heidelberg, Germany; ^3^ Department of General Psychiatry, University Hospital Heidelberg, University of Heidelberg, Heidelberg, Germany

**Keywords:** complex PTSD, cognitive functioning, visual memory, executive functioning, attention

## Abstract

**Background:**

Previous research has indicated cognitive impairments in patients with post-traumatic stress disorder (PTSD), specifically in attention, memory, and executive functioning. However, there is limited knowledge about the cognitive profile of individuals with complex PTSD (cPTSD), a new diagnosis in ICD-11. Moreover, predictors of cognitive impairment remain unclear. The present study aims to enhance understanding of cognitive functioning and its predictors in cPTSD compared with non-complex PTSD (ncPTSD).

**Methods:**

*N* = 64 participants (*n* = 34 cPTSD, *n* = 30 ncPTSD) completed psychometric questionnaires and the neuropsychological test set Cognitive Basic Assessment (COGBAT) assessing a general cognitive index, attention, visual memory, and executive functioning. First, the test results of both groups were compared to the COGBAT norm sample. Secondly, group differences in cognitive domains were analyzed using student *t*-tests with independent samples (cPTSD vs. ncPTSD). Thirdly, bivariate and multivariate regressions examined influencing factors of cognitive impairment.

**Results:**

Both groups showed cognitive impairments in comparison to the COGBAT norm group. Significant differences between cPTSD and ncPTSD were found in visual memory (*p* = .003) and selective attention (*p* = .004). In multivariate regression, type of PTSD and age were found to significantly impact visual memory, while type of PTSD, age, and psychotropic medication showed significant effects on selective attention.

**Conclusions:**

Given higher symptom severity and cognitive deficits in cPTSD, more intensive and diverse interventions should be considered in comprehensive treatment plans, for instance, cognitive training.

## 1 Introduction

Experiencing traumatic events can significantly impact mental well-being, potentially leading to mental disorders. In the 11th version of the International Classification of Diseases (ICD-11), the diagnosis of post-traumatic stress disorder (PTSD) is complemented by the new diagnosis of complex post-traumatic stress disorder (cPTSD), acknowledging the diverse symptomatology resulting from trauma (1, 2). In addition to the PTSD symptom triad of re-experience, avoidance, and hyperarousal, the diagnosis of cPTSD includes disturbances in self-organization (DSO): negative self-concept, interpersonal problems, and affective dysregulation. There is an ongoing debate on whether cPTSD is a distinct diagnosis from the “classic” PTSD, which will be referred to as “non-complex” PTSD (ncPTSD) in the following. In contrast to ICD-11, the new diagnosis of cPTSD was not included in the fifth version of the Diagnostic and Statistical Manual of Mental Disorders (DSM-5; 3). However, in DSM-5, the diagnostic criteria for PTSD were expanded to include negative changes in mood and cognition as well as a dissociative subtype. The different symptom profiles resulting from this expansion of diagnostic criteria are criticized for complicating treatment planning based on the diagnosis (1). Overall, recent studies and clinical observations emphasize the need for both diagnostic entities (cPTSD and ncPTSD) (4–6). Moreover, cPTSD and ncPTSD result in different therapeutic strategies based on symptom profiles (1, 7). Current data from a representative German sample shows a one-month prevalence of 0.5% for cPTSD compared to 1.5% for ncPTSD (8). Given the relative novelty of this research field, further research is needed to enhance our understanding of cPTSD and improve therapeutic approaches.

Previous research indicates that trauma-related disorders are often associated with impairments in cognitive performance, even for emotionally neutral stimuli (9–11). In total, research on cognitive functioning in patients with trauma-related disorders identified cognitive impairments, primarily in the domains of memory, attention, and executive functioning (9, 10, 12–14), especially in tasks that require processing speed (11). From a clinical standpoint, these impairments may be highly relevant, as cognitive functioning was shown to play a role in coping with posttraumatic stress (10–12, 15). In previous research consisting of neuropsychological assessments, several factors have been identified to impair cognitive performance in patients with trauma-related disorders, including clinical variables such as higher posttraumatic symptom severity, dissociation, and comorbidities, such as depression and anxiety (9, 11, 16–18). Moreover, sociodemographic factors showed a significant influence on cognition in PTSD, with higher age and male gender being associated with lower cognitive performance (9, 10). In addition, social support was shown to be associated with better cognition, a factor known to buffer traumatic experiences and to protect against the development of posttraumatic stress (19, 20). However, most studies on cognition in trauma patients have not differentiated between cPTSD and ncPTSD, potentially resulting in heterogeneous populations varying in posttraumatic symptom profiles.

To our knowledge, only a few studies have investigated the differences between patients with cPTSD and ncPTSD regarding cognitive performance. Shin et al. (2021) investigated emotional perception, visual attention, and working memory in adolescents with cPTSD or ncPTSD (21). They found that the cPTSD group had more deficits in all cognitive functions when compared to the ncPTSD group. In addition, the cognitive test results in emotional perception, visual attention, and working memory correlated with the severity of cPTSD symptoms. Biscoe et al. (2024) examined executive functioning in veterans with cPTSD and ncPTSD (22). Their results showed an association between cognitive impairments and DSO symptoms. As these studies examined only a few, isolated cognitive functions in specific samples (i.e., adolescents and veterans), we aimed to explore a broad range of cognitive domains in patients with cPTSD compared to patients with ncPTSD. Further, we sought to examine potential influencing factors regarding cognitive functioning. We used a comprehensive and validated test set to assess cognitive functioning. This test set offers a standardized norm sample, allowing the comparison of the cognitive performance of patients with cPTSD and ncPTSD with a representative sample (23).

Considering previous research findings of cognitive functioning in trauma-exposed patients (9–14) as well as the studies on specific cognitive impairments in cPTSD (21, 22), we hypothesized that patients with cPTSD display more severe impairment in the cognitive domains of attention, memory, and executive function, compared to patients with ncPTSD. Regarding factors influencing cognitive impairments in trauma patients (cPTSD and ncPTSD), we hypothesized that symptom severity, age, gender, the presence of depression, anxiety, and dissociation are negatively associated with cognitive performance, whereas the presence of social support is positively associated with cognitive performance.

## 2 Methods

### 2.1 Participants and procedure

This study was conducted at the University Hospital in Heidelberg, Germany, from June 2021 to November 2023. German-speaking patients of the psychotraumatology outpatient clinic of at least 18 years of age with a clinical diagnosis of cPTSD or ncPTSD were informed about the study by telephone. In addition, patients were recruited via press coverage and flyers at psychotherapists’ practices. Exclusion criteria were severe psychiatric comorbidities, such as psychosis, bipolar disorder, or substance dependence as well as histories of neurological disorders or traumatic brain injury.

**Figure 1:**
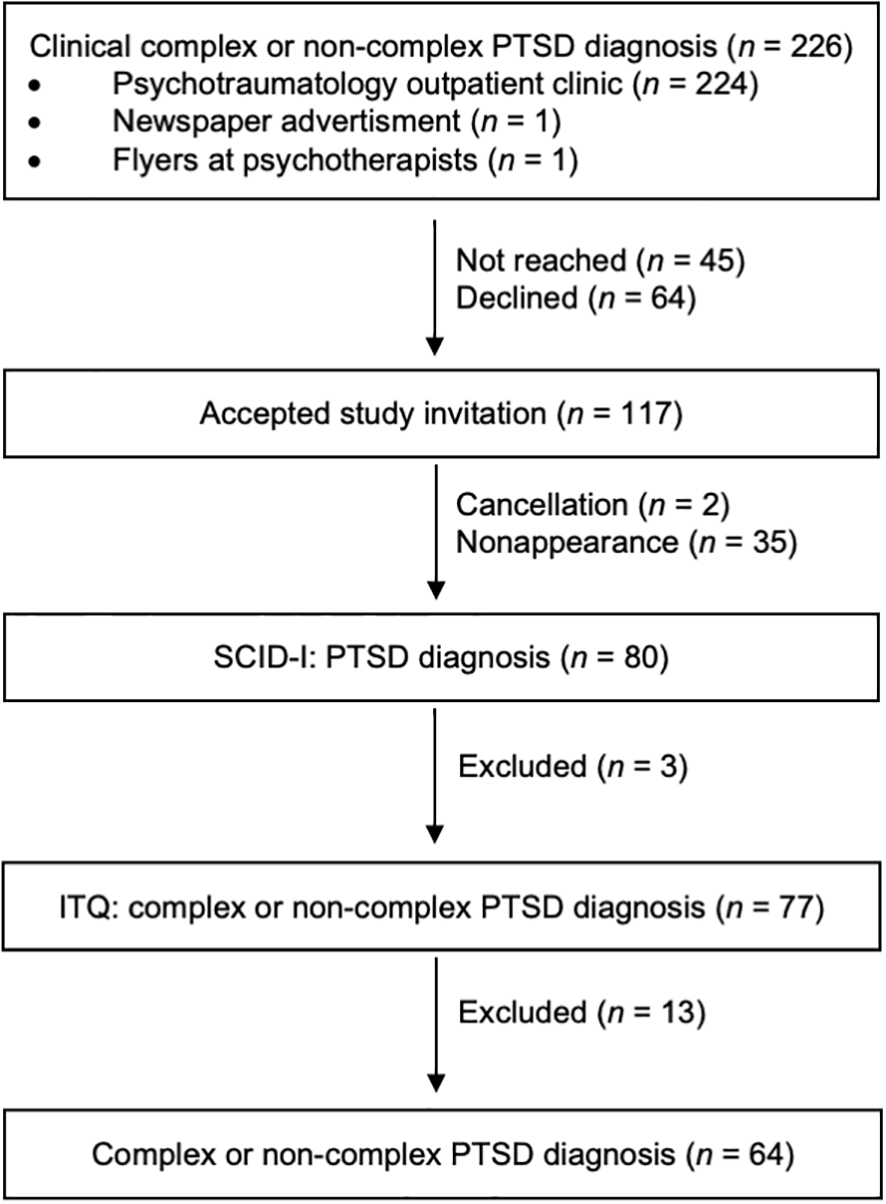
Flow diagram of recruitment and three stages of diagnostic process. PTSD, posttraumatic stress disorder, SCID-I, Structured Clinical Interview for DSM-IV (24), ITQ, International Trauma Questionnaire (25).

The recruitment process (**Figure 1**) involved a three-stage assessment to clinically confirm PTSD diagnosis and differentiate between cPTSD and ncPTSD. (1.) Individuals clinically diagnosed with cPTSD or ncPTSD (*N* = 226) were invited to participate. Of these, *n* = 117 accepted the invitation, resulting in a response rate of 52%. (2.) The PTSD section of the *Structured Clinical Interview for DSM-IV for Axis 1* (SCID-I, 24) ensured the current presence of the diagnosis, and, (3.) using the *International Trauma Questionnaire* (ITQ, 25), group allocation was defined. *N* = 64 participants met diagnostic criteria for cPTSD (*n* = 34) or ncPTSD (*n* = 30) and were included in the analysis.

After agreeing to participate, patients received the study material by post, including an information letter about the study, the written consent form, and the psychometric questionnaires. After, the in-person assessment was conducted, using the SCID-I, the *Multiple Choice Vocabulary Test* (MWT-B, 26), and the *Cognitive Basic Assessment test set* (COGBAT, 27). An overview of the assessment material is shown in **Table 1**. The study duration was a maximum of three hours. For compensation, participants received 20 euros.

**Table 1:** Assessment material.

Diagnostic tool	Abbreviation (author)
Psychometric Assessment	
Generalized Anxiety Disorder 7 Questionnaire	GAD-7 (Spitzer et al., 2006) (31)
German version of Dissociative Experience Scale	FDS-20 (Spitzer et al., 2004) (34)
International Trauma Questionnaire	ITQ (Cloitre et al., 2018) (25)
Patient Health Questionnaire, Depression Module	PHQ-9 (Kroenke & Spitzer, 2002) (29)
Perceived Social Support Questionnaire	F-SozU K-14 (Fydrich et al., 2009) (35)
Posttraumatic Stress Disorder Scale	PDS (Ehlers et al., 1996; Foa, 1995) (38, 39)
Structured Clinical Interview for DSM-IV	SCID-I (Wittchen et al., 1997) (24)
Trauma Symptom Inventory	TSI-2 (Briere, 2011) (41)
Cognitive Assessment	
Multiple Choice Vocabulary Test (Version B) ^1^	MWT-B (Lehrl, 2005) (26)
Cognitive Basic Assessment test set ^2^	COGBAT (Aschenbrenner et al., 2012) *^a^* (27)
Attention	
Perception and Attention Functions: Alertness	WAF-A (Sturm, 2006) (47)
Perception and Attention Functions: Divided Attention	WAF-G (Sturm, 2006) (47)
Trail Making Test – L Version A: Processing Speed	TMT-A (Rodewald et al., 2012) (48)
Figural Long-Term Memory	
Figural Memory Test: Learning and Memory	FGT (Vetter et al., 2012) (49)
Executive functioning	
N-Back Verbal Test: Verbal Working Memory	NBV (Schellig & Schuri, 2009) (50)
Tower of London-Freiburg Version: Planning Ability	TOL-F (Kaller et al., 2011) (51)
Trail Making Test – L Version B: Cognitive Flexibility	TMT-B (Rodewald et al., 2012) (48)
Response Inhibition Test: Inhibition	INHIB (Kaiser et al., 2010) (52)

a Vienna Test System Schuhfried GmbH*;* ^1^paper-pencil-based assessment, ^2^computer-based assessment.

### 2.2 Ethics approval and consent to participate

The study protocol was developed according to the Helsinki II declaration (28). The Ethics Committee of the Medical Faculty of the University of Heidelberg approved the study under file number S-210/2020. Written informed consent was obtained from all study participants.

### 2.3 Measures

#### 2.3.1 Psychometric assessment

Depression was assessed with the *Patient Health Questionnaire, Depression Module* (PHQ-9, 29) of the German version of the *Patient Health Questionnaire* (PHQ-D, 30). To measure anxiety, we used the German version of the anxiety module *Generalized Anxiety Disorder 7* (GAD-7, 31) of the PHQ-D. German translations of the PHQ-9 and GAD-7 showed good internal consistency with Cronbach´s α = .85-.88 (32, 33). Dissociative symptoms were assessed using the FDS-20, the German translation and shortened screening version of the *Dissociative Experience Scale*, which showed good to excellent internal consistency with Cronbach´s α = .89-.93 (34). To evaluate perceived social support, we used the German questionnaire F-SozU K-14 with excellent internal consistency (Cronbach`s α = .94) (35).

As previously mentioned, we used the German version of the ITQ (25) for group assignment in order to differentiate between cPTSD and ncPTSD. The first section focuses on the three core symptoms of PTSD, with two items used to evaluate each symptom. Participants rate how severely they have been affected by the symptoms in the last month. The second section measures disturbances in self-organization (DSO) symptoms, consisting of three symptom domains (negative self-concept, interpersonal problems, and affective dysregulation). Participants answer the DSO items regarding how much these apply to them or their interactions with others in the past month. In addition, there are three questions on functional impairment in important areas of life for the PTSD and DSO sections. A diagnosis of ncPTSD requires a score of two or higher in at least one item of each PTSD symptom domain and functional impairment. A diagnosis of cPTSD requires fulfillment of the ncPTSD criteria and a score of two or higher on at least one item in each DSO symptom domain and functional impairment. The ITQ has satisfactory to excellent internal consistency (Cronbach’s *α* = .63 -.93) (6, 36, 37).

The *Posttraumatic Stress Disorder Scale* (PDS) is a screening instrument for the presence of PTSD according to the diagnostic criteria of the DSM-IV. We used the German translation (38, 39). In the present study, it was used to assess classic PTSD symptoms (re-experience, avoidance, and hyperarousal), in particular symptom severity. We used the list of traumatic events, or in cases of multiple traumas, the most distressing one, to determine the type of trauma. The PDS has excellent internal consistency with Cronbach’s α = .92 (40). In addition, the German version of the *Trauma Symptom Inventory* (TSI-2, 41) was administered to assess a broad range of posttraumatic symptoms with 136 items. Moreover, TSI-2 was applied to measure symptoms of the DSO cluster, using the following TSI-2 scales: inadequate self-reference to determine the negative self-image, insecure attachment to assess interpersonal problems, and the mean value of the scales depression, dissociation, anger, and tension reduction behavior to assess affect dysregulation (42). Most scales of the TSI-2 have an acceptable to excellent internal consistency (Cronbach’s *α* = .62 -.95) (43).

#### 2.3.2 Cognitive testing

The MWT-B (26), a German questionnaire, was administered to control for premorbid intelligence deficits. It consists of 37 items, each containing five words, including four nonsense words. Participants are asked to cross out the word that exists in the German language for each item. The test results correlate strongly with the IQ of healthy adults (44). For a comprehensive evaluation of cognitive dimensions (i.e., attention, memory, and executive function), we used the German version COGBAT of the Vienna test system of Schuhfried GmbH (27). The COGBAT consists of six standardized tests administered on a computer using a mouse and a special keyboard lasting approximately 60 minutes. The norm sample consists of *N* = 419 people aged 16 to 80 years and allows a comparison with specific norms. The test battery has been validated in different populations (45, 46). The internal consistencies of Cronbach’s *α* >.70 are acceptable to excellent for all tests (23). The variables were selected based on the standard results protocol and COGBAT manual. The COGBAT tests employed in this study include the following:

*Attention:* Selective attention is measured using the test for *Perception and Attention Functions - Alertness* (WAF-A, 47). The task is to react as fast as possible to a presented stimulus. Divided attention is measured using the *Perception and Attention Functions - Divided Attention* (WAF-G, 47). The participants are presented with visual (squares) and auditory (sounds) stimuli simultaneously. They are asked to press a button if any type of stimulus changes twice in a row. Processing speed is measured using the *Trail Making Test – Part A* (TMT-A, 48) by connecting numbers as fast as possible in ascending order.

*Visual memory:* Visual learning and memory are measured using the *Figural Memory Test* (FGT, 49). Figures are presented in a learning phase for five consecutive times. This is followed by a short-term delayed (5-minute interval) and long-term delayed (30-minute interval) recall. Lastly, a recognition phase follows, in which the previously learned figures need to be discriminated from similar, but unknown figures.

*Executive functioning:* Verbal working memory is assessed using the *N-Back Verbal Test* (NBV, 50), wherein letters are presented one after the other in a 2-back paradigm. Planning ability is measured using the *Tower of London-Freiburg Version* test (TOL-F, 51). Participants are asked to rearrange three different colored balls to match a presented target state using the fewest possible steps, adhering to predetermined rules. Points are given for the correct solution achieved with the minimum number of steps within a 60-second time frame. The *Trail Making Test – Part B* (TMT-B, 48) assesses cognitive flexibility by connecting numbers and letters in alternating ascending order as fast as possible. Response inhibition is assessed using the *Response Inhibition Test* (INHIB, 52) using a Go-NoGo-paradigm.

### 2.4 Statistical analysis

The data were analyzed using the “Statistical Package of Social Sciences” (SPSS; IBM SPSS Statistics for Macintosh, Version 29.0) (53). A power analysis was performed *a priori* with G*Power (version 3.1) (54) to estimate the sample size. In previous research, effect sizes on cognitive impairments in PTSD were dependent on the control group and the domain (10). No prior effect sizes were found for differences in cognition between cPTSD and ncPTSD. For a *t*‐test (one‐tailed) for two independent samples with an effect size of *d* = 0.70 (*α* = 0.05, *power* = 0.80, allocation ratio *N2/N1 =* 1), a total sample size of *N* = 52 was needed. Therefore, the current study has the power to detect large effect sizes.

The sociodemographic and psychometric parameters of the cPTSD and ncPTSD groups are presented using descriptive statistics. Group differences (cPTSD vs. ncPTSD) were tested for significance using student *t*-tests for independent samples or *χ^2^*-tests. For descriptive analyses, *p*-values of <.05 (one-tailed) were considered statistically significant.

To assess objective cognitive impairment of cPTSD and ncPTSD, we used percentile ranks comparing cognitive scores to a representative, healthy COGBAT norm sample based on age (16-30 years *n* = 127, 31-50 years *n* = 151, 51-80 years *n* = 141) (23). For the group comparison between cPTSD and ncPTSD concerning cognitive functioning, the raw scores of each individual were converted into standardized *z*-scores. If necessary, scores were inverted for higher scores describing better cognitive performance. A group comparison was performed using student *t*-tests for independent samples. First, we tested the composite scores for each domain (attention, visual memory, and executive functioning), with the subdomains also being analyzed if the domain scores were not significantly different. Bonferroni correction was applied on one-tailed *p* values to minimize Type I errors (11-test family, corrected *p* <.005). ANCOVAs were performed to control for the covariate medication. Additionally, we calculated bivariate regression analyses for cognitive functions with significant group differences between cPTSD and ncPTSD with all explanatory variables. Bonferroni correction was applied on one-tailed *p* values (12-test family, corrected *p* <.004). Lastly, we performed multivariate linear regressions to investigate predictors of cognitive impairment (i.e. PTSD type, age, cPTSD symptoms, PTSD symptoms, depression, anxiety, dissociation, and social support).

## 3 Results

### 3.1 Demographic and clinical assessment

**Table 2** shows the participants’ sociodemographic and clinical characteristics. The age range in the cPTSD group was 20 - 65 years (*median* = 43.50, *IQR* = 25.25). In the ncPTSD group, age ranged from 20 to 67 years (*median* = 33.50, *IQR* = 32.50). In both groups, most participants were female. The groups did not differ significantly in age, gender distribution, or premorbid intelligence level. Individuals in the cPTSD group reported to have children (*p* = .041; cPTSD: *M* = 1.24 children (*SD* = 1.33) more often than the ncPTSD group: *M* = 0.70 children (*SD* = 1.06)). No significant differences were observed between groups in other sociodemographic or clinical variables. Regarding the type of trauma, in the cPTSD group, *n* = 4 had experienced an accident, *n* = 4 violence, *n* = 15 sexual assault, *n* = 1 war, and *n* = 1 a life-threatening illness; *n* = 9 reported other traumatic experiences. In the ncPTSD group, *n* = 6 individuals had experienced an accident, *n* = 1 a natural disaster, *n* = 10 violence, *n* = 2 sexual assault, *n* = 1 captivity, *n* = 1 a life-threatening illness, and *n* = 9 other traumatic events.

**Table 2:** Sociodemographic and clinical assessment of cPTSD and ncPTSD group.

Continuous variables, *M (SD)*	ncPTSD	cPTSD	Test statistics	
	*n* = 30	*n* = 34	*t*	*p*
Age (years)	40.67 (16.15)	42.24 (13.06)	0.42	.674
MWT-B	29.20 (3.95)	28.74 (3.74)	-0.48	.631
Categorial variables, *n (%)*			χ^2^	Asym. Sign.
Sex (female)	24 (80)	26 (76)	0.12	.733
Native language (german)	28 (93)	32 (94)	0.02	.897
Nationality (german)	29 (96)	33 (97)	0.01	.928
Physical complaints	24 (80)	29 (85)	0.31	.575
Children (yes)	10 (33)	20 (59)	4.16	.041*
Employed	18 (60)	17 (50)	0.64	.423
Educational level			1.82	.610
9 - 10 years	5 (16)	10 (29)		
10 - 12 years	13 (43)	13 (38)		
12 - 13 years (A-Level)	8 (26)	6 (18)		
Higher Education	4 (13)	5 (15)		
Medication				
Antidepressants	16 (53)	18 (53)	0.00	.975
Antipsychotics	1 (3)	6 (18)	3.35	.067
Benzodiazepines	1 (3)	2 (6)	.232	.630
Previous Psychotherapy				
Outpatient Therapy	23 (76)	31 (91)	2.55	.111
Inpatient Therapy	19 (63)	28 (82)	2.96	.086
Family status			7.71	.103
Single	8 (26)	13 (38)		
Relationship	11 (36)	4 (12)		
Married	8 (26)	11 (32)		
Others	2 (6)	1 (3)		
Housing situation ^a^			4.93	.177
Living Alone	6 (21)	14 (41)		
With partner	11 (38)	6 (18)		
Shared flat	3 (10)	2 (6)		
With family	9 (31)	12 (35)		

cPTSD, complex post-traumatic stress disorder; ncPTSD, non-complex post-traumatic stress disorder; MWT-B, Multiple Choice Vocabulary Test (26); *significant at *p* <.05; ^a^ncPTSD *n* = 29.

### 3.2 Psychometric assessment

The mean and standard deviations of self-reported psychometric assessment are shown in **Table 3**. The severity of symptoms was significantly higher in cPTSD group than in the ncPTSD group regarding the following domains with medium to large effect sizes: PTSD symptom sum score (ITQ) (*d* = 0.58), DSO symptoms sum score (ITQ) (*d* = 2.05), PTSD symptom severity (PDS) (*d* = 0.90), cPTSD symptom severity (TSI) (*d* = 1.06), dissociation (FDS-20) (*d* = 0.87), depression (PHQ-9) (*d* = 1.29), anxiety (GAD-7) (*d* = 0.62), depression (TSI) (*d* = 1.02), dissociation (TSI) (*d* = 0.85), somatic preoccupations (TSI) (*d* = 0.66), sexual disturbances (TSI) (*d* = 0.69), suicidality (TSI) (*d* = 1.22), insecure attachment (TSI) (*d* = 0.82), impaired self-reference (TSI) (*d* = 0.94), tension reduction behavior (TSI) (*d* = 0.76), the factor somatization (*d* = 0.63), self-disturbance (*d* = 1.06), posttraumatic stress (*d* = 0.66), and externalization (TSI) (*d* = 0.95). A significant difference with a small effect size was found for anxious arousal (TSI) (*d* = 0.44), defensive avoidance (*d* = 0.44), with a higher score in the cPTSD group (all *p* <.05). Moreover, the cPTSD group showed significantly less social support (FSozU K-14) with a medium effect size (*d* = -0.76, *p* <.05). No significant group differences were found for PTSD symptoms sum score (ITQ), anxiety (GAD-7), somatic preoccupations (TSI), anxious arousal (TSI), anger (TSI), intrusive experiences (TSI), defensive avoidance (TSI), and factors of somatization and posttraumatic stress (TSI).

**Table 3:** Psychometric assessment of cPTSD and ncPTSD group.

	ncPTSD	cPTSD	Test statistics	
	*n* = 30	*n* = 34	*t*	*p*
ITQ sum score				
PTSD symptoms	17.00 (3.17)	18.97 (3.55)	2.33,	.012*
DSO symptoms	9.67 (3.61)	17.24 (3.77)	8.18	<.001**
Symptom severity PTSD (PDS)	29.77 (7.39)	36.12 (6.77)	3.60	<.001**
Symptom severity cPTSD (TSI-2) ^a^	31.57 (14.67)	48.27 (16.54)	4.21	<.001**
Dissociation (FDS-20) ^a^	2.01 (1.91)	3.80 (2.18)	3.45	<. 001*
Depression (PHQ-9)	12.97 (3.62)	18.18 (4.38)	5.14	<.001**
Anxiety (GAD-7)	11.43 (4.07)	13.85 (3.69)	2.49	.008*
TSI (*T*-Scores) ^a^				
Anxious Arousal	61.93 (8.85)	65.53 (7.71)	1.73	.045*
Depression	58.66 (8.73)	61.93 (8.85)	4.03	<.001**
Anger	56.45 (12.09)	57.26 (12.61)	0.26	.397
Intrusive Experiences	69.03 (9.85)	72.32 (11.07)	1.24	.111
Defensive Avoidance	62.03 (8.01)	66.03 (9.78)	1.75	.042*
Dissociation	54.21 (10.84)	66.26 (16.48)	3.48	<.001**
Somatic Preoccupations	54.97 (12.76)	62.73 (10.01)	2.62	.006*
Sexual Disturbance ^c^	54.10 (10.55)	61.94 (11.94)	2.70	.004*
Suicidality	50.64 (6.97)	68.24 (18.55)	5.11	<.001**
Insecure Attachment	61.50 (10.02)	50.64 (6.97)	3.26	<.001**
Impaired Self-Reference	52.21 (11.75)	63.12 (12.14)	3.72	<.001**
Tension Reduction Behavior	53.00 (11.25)	64.62 (17.91)	3.13	<.001**
TSI Factors ^a^				
Somatization	55.66 (11.69)	62.47 (10.01)	2.49	.008*
Self-Disturbances	55.64 (7.92)	64.15 (8.10)	4.13	<.001**
Posttraumatic Stress	63.66 (7.36)	69.15 (9.05)	2.61	.006*
Externalization	54.28 (7.94)	63.66 (7.36	3.77	<.001**
Social support (F-SozU) ^b^	3.84 (0.86)	3.21 (0.77)	-2.99	.002*

cPTSD, complex post-traumatic stress disorder; ncPTSD, non-complex post-traumatic stress disorder; ITQ, International Trauma Questionnaire (25); DSO, Disturbances in self-organization; PDS, Posttraumatic Stress Disorder Scale (38, 39); PHQ-9, Patient Health Questionnaire, Depression Module (29); GAD-7, Generalized Anxiety Disorder 7 Questionnaire (31); FDS-20, German version of Dissociative Experience Scale (34); TSI-2, Trauma Symptom Inventory (41); F-SozU, Perceived Social Support Questionnaire (35); presentation as mean and standard deviation; * significant at *p* <.05, ** significant at *p* <.001; *^a^* ncPTSD *n* = 29, ^b^ncPTSD *n* = 28, *^c^* cPTSD *n* = 32.

### 3.3 Cognitive assessment

**Table 4** presents percentile ranks of cognitive performance on a dimensional level for the cPTSD and ncPTSD groups compared to an age-matched comparison group derived from the representative norm sample of the COGBAT. Across all domains, the cPTSD group remained below the 16th percentile in 21 – 44% of the test scores, whereas the ncPTSD group remained below the 16th percentile in 7 – 14% of the test scores, indicating significant cognitive impairment in both patient groups compared to the norm sample.

**Table 4:** Neuropsychological assessment: Percentile ranks of cPTSD and ncPTSD group compared to an age-matched COGBAT norm sample.

Dimension, *n (%)*	Percentile ncPTSD (*n* = 29)				Percentile cPTSD (*n* = 34)			
	≤ 15	16 - 24	25 - 75	> 75	≤ 15	16 - 24	25 - 75	> 75
COGBAT Index	2 (7)	4 (8)	17 (59)	6 (21)	15 (44)	3 (9)	12 (35)	4 (12)
Attention	2 (7)	0 (0)	19 (66)	8 (28)	7 (21)	1 (3)	21 (62)	5 (15)
Visual memory	3 (10)	6 (21)	11 (38)	9 (31)	14 (41)	5 (15)	12 (35)	3 (9)
Executive functioning	4 (14)	3 (10)	19 (66)	3 (10)	11 (32)	2 (6)	17 (50)	4 (12)

cPTSD, complex post-traumatic stress disorder; ncPTSD, non-complex post-traumatic stress disorder; *COGBAT*, Cognitive Basic Assessment test set (27); percentile rank *≤* 15 = cognitive impairment, below average range; percentile rank 16 - 24 = possible impairment, low average range; percentile rank 25 - 75 = no impairment, average range; > 75 = no impairment, above average range.

**Table 5** shows the comparison of COGBAT scores for the cPTSD and the ncPTSD group. Significant differences with medium effect sizes were found for visual memory (*d* = -0.73) on a cognitive domain level, showing lower cognitive performance in the cPTSD group. The groups did not significantly differ in executive functioning or attention domains. On a subdomain level, a significant difference between groups with medium effect sizes was found in selective attention (reaction time) (*d* = -0.70), with lower cognitive performance of cPTSD group. No significant group differences were found for working memory, planning ability, cognitive flexibility, inhibition, and divided attention (mean reaction time). After adjusting for medication, results on visual memory and selective attention remained significant.

**Table 5:** Neuropsychological assessment: Dimensional scores and test scores of complex post-traumatic stress disorder (cPTSD) and non-complex post-traumatic stress disorder (ncPTSD) group.

	ncPTSD	cPTSD	Test statistics	
	*n* = 30	*n* = 34	*t*	*p*
COGBAT Index ^a^	0.08 (2.99)	-2.24 (4.32)	-2.51	.007
Attention ^a^	0.17 (0.85)	-0.28 (1.33)	-1.58	.060
WAF-A: Selective Attention (z score)	0.35 (0.62)	-0.31 (1.17)	-2.79	.004*
WAF-G: Divided Attention ^b^ (z score)	0.17 (0.93)	-0.15 (1.05)	1.30	.102
TMT A: Processing speed (z score)	0.08 (0.809	-0.07 (1.16)	-0.61	.271
FGT: Visual memory ^a^	-0.05 (3.34)	-2.71 (3.91)	-2.87	.003*
Executive functioning ^a^	-0.06 (0.91)	-0.39 (1.38)	-1.10	.139
NBV: Working memory (z score)	-0.03 (0.97)	0.02 (1.04)	0.21	.418
TOL: Planning ability (zscore)	0.09 (0.95)	-0.08 (1.05)	-0.66	.256
TMT-B: Cognitive flexibility (z score)	0.18 (0.66)	-0.16 (1.21)	-1.38	.087
*INHIB: Inhibition (z score)*	0.05 (0.84)	-0.13 (1.12)	-1.13	.130

cPTSD, complex post-traumatic stress disorder; ncPTSD, non-complex post-traumatic stress disorder; COGBAT, Cognitive Basic Assessment test set (27), WAF-A, Perception and Attention Functions: Alertness (47); WAF-G, Perception and Attention Functions: Divided Attention (47); TMT A, Trail Making Test – L Version A (48); FGT, Figural Memory Test (49); NBV, N-Back Verbal Test (50); TOL, Tower of London (51); TMT - B Trail Making Test – L Version B (48); INHIB, Response Inhibition Test (52); variables in italics are not included in the dimensional scores; presentation as mean and standard deviation; * significant at Bonferroni corrected: *p* <.005; *^a^* ncPTSD *n* = 29, *^b^* cPTSD *n* = 28.

### 3.4 Influencing factors of cognitive impairments

**Table 6** shows bivariate regressions for selective attention and visual memory with type of PTSD (cPTSD vs. ncPTSD), age, gender, cPTSD symptoms, PTSD symptoms, depression, anxiety, dissociation, and social support. In the bivariate regressions, after Bonferroni correction, higher age (*β* = -548, *p* <.001) was the only factor to remain significant, being associated with lower performance in visual memory.

**Table 6:** Bivariate regressions of cognitive impairments.

	Visual memory		Selective Attention	
	*Beta*	*p*	*Beta*	*p*
PTSD type ^a^	.345	.006	.334	.007
Age	-.548	<.001*	-.317	.011
Gender (female/male)	.259	.041	.291	.018
Sexual trauma (no/yes)	-.191	.134	-.01	937
Psychotherapy (no/yes) ^b^	-.162	.205	-.008	.947
Medication (no/yes) ^c^	-.175	.171	.255	.042
cPTSD symptoms (TSI)	.067	.604	-.041	.747
PTSD symptoms (PDS)	-.038	.766	-.12	.344
Depression (PHQ-9)	-.284	.024*	-.026	.05
Anxiety (GAD 7)	-.315	.012*	-.104	.416
Dissociation (FDS-20)	.023	.862	-.125	.328
Social support (F-SozU)	.105	.415	.057	.661

*Beta* = standardized regression weights; ^a^ PTSD type (1 = cPTSD, 2 = ncPTSD), *^b^* Psychotherapy (inpatient and/or outpatient), *^c^* Medication (antidepressants, antipsychotics and/or benzodiazepines), TSI = Trauma Symptom Inventory (41), PDS = Posttraumatic Stress Disorder Scale (38, 39), PHQ-9 = Patient Health Questionnaire, Depression Module (29); GAD-7 = Generalized Anxiety Disorder 7 Questionnaire (31), FDS = German version of Dissociative Experience Scale (34), F-SozU K14 = Perceived Social Support Questionnaire (35); * significant at Bonferroni corrected *p* <.004.

**Table 7** shows the results of multivariable omnibus regression. For the visual memory, the model explains 54.1% of the variance (*R^2^* = 54.1, *adj*. *R^2^* = 42.9, *F* (8, 53) = 4.82, *p* <.001), with cPTSD group (*β* = .313, *p* = .021) and age (*β* = -.459, *p* <.001) negatively influencing cognitive performance. For selective attention, the model explains 40.5% (*R^2^* = 40.5, *adj*. *R^2^* = 25.9, *F* (8, 53) = 2.78, *p* = .006) of the variance, with cPTSD group (*β* = .302, *p* = .049), age (*β* = -.321, *p* =.022) negatively influencing cognitive performance and psychotropic medication (*β* = .298, *p* = .018) positively influencing selective attention.

**Table 7:** Multiple regressions of cognitive impairments.

	Visual memory		Selective attention	
	*Beta*	*p*	*Beta*	*p*
PTSD type ^a^	.313	.021*	.302	.049*
Age	-.459	<.001**	-.321	.022*
Gender (female/male)	.088	.424	.202	.109
Sexual trauma (no/yes)	-.196	.101	.056	.678
Psychotherapy (no/yes) ^b^	.037	.732	.016	.895
Medication (no/yes) ^c^	-.176	.105	.298	.018*
cPTSD symptoms (TSI)	.290	.064	.057	.745
PTSD symptoms (PDS)	.059	.649	-.028	.852
Depression (PHQ-9)	-.043	.796	-.208	.272
Anxiety (GAD 7)	-.274	.066	.094	.573
Dissociation (FDS-20)	-.036	.825	-.096	.603
Social support (F-SozU)	-.086	.467	-.167	.217

*Beta* = standardized regression weights; ^a^ PTSD type (1 = cPTSD, 2 = PTSD); *^b^* Psychotherapy (inpatient and/or outpatient), *^c^* Medication (antidepressants, antipsychotics and/or benzodiazepines), TSI-2 = Trauma Symptom Inventory (41), PDS = Posttraumatic Stress Disorder Scale (38, 39), PHQ-9 = Patient Health Questionnaire, Depression Module (29); GAD-7 = Generalized Anxiety Disorder 7 Questionnaire (31), FDS-20 = German version of Dissociative Experience Scale (34), F-SozU K14 = Perceived Social Support Questionnaire (35); * significant at *p* <.05, ** significant at *p* <.001.

## 4 Discussion

The present study examined cognitive functioning in patients with cPTSD and ncPTSD using a three-stage diagnostic process and a standardized neuropsychological test set. In general, both patient groups showed objective cognitive impairment compared to a healthy norm sample. The cPTSD group performed significantly worse in the cognitive testing than the ncPTSD group, particularly in selective attention and visual memory. Moreover, age and type of PTSD (cPTSD vs. ncPTSD) were identified to be associated with cognitive performance in patients suffering from posttraumatic stress.

### 4.1 Symptom profiles in cPTSD and ncPTSD

With regard to the classic PTSD symptoms of re-experience, avoidance, and hyperarousal, the results of the group comparison showed a significant difference, with a higher symptom load in the cPTSD group. Further analysis of posttraumatic symptoms applying the TSI showed that cPTSD patients displayed significantly higher levels of defensive avoidance, somatic preoccupations, sexual disturbance, suicidality, insecure attachment, greater impairments in self-reference, and more frequent use of tension reduction behavior. These results are in line with previous findings, indicating that patients with cPTSD exhibit a generally higher symptom load compared to ncPTSD (1, 5, 55, 56). Furthermore, our findings were consistent with previous studies, demonstrating that patients with cPTSD suffer from higher depressive symptom severity (36, 57), more severe dissociation (58, 59), and report less social support (60). We did find a significant group difference in anxiety symptoms. Even though cPTSD was associated with higher levels of anxiety in a large sample of a study by Karatzias et al. (2019) (57), recent studies with comparable sample sizes to our study did not find significant differences in anxiety between cPTSD and ncPTSD (21, 61). Concerning our finding of higher symptom severity in cPTSD, it can be assumed that cPTSD may generally be associated with disruptions in essential psychological capacities, e.g. coping strategies, some of which manifest in DSO symptoms (1). These disruptions, in turn, may complicate the processing of comorbid symptomatology like depression or dissociation, resulting in higher symptom severity for patients with cPTSD. Regarding type of trauma, the majority of cPTSD patients (44%) reported sexual assault as their most distressing trauma, compared to only 0.6% in the ncPTSD group. This is in line with previous research that showed that interpersonal trauma, especially sexual assault, is strongly associated with psychological dysfunction in general and with a higher risk to result in PTSD than non-interpersonal trauma (62–64).

### 4.2 Cognitive impairments in cPTSD and ncPTSD

In our study, norm group comparison indicated that both patient groups showed cognitive impairments in all cognitive domains. Specifically, in the domain of visual memory, we observed significant group differences, indicating lower cognitive performance of the cPTSD group compared to the ncPTSD group. In addition to studies showing verbal memory deficits in patients with PTSD (13, 14), further studies found impaired non-verbal memory to be linked with PTSD (65–67). Our findings align with prior research, demonstrating that higher PTSD symptom load is associated with worse performance in visual memory (66). Previous studies discussed reduced hippocampal volume as a possible reason for these impairments in memory (65). The underlying assumption is that stress induces changes in the hippocampus, which results in a reduced volume. Consequently, the severe traumatic experiences and higher symptom burden linked to cPTSD could lead to greater stress levels, thereby contributing to more pronounced reductions in hippocampal volume and, subsequently, more significant impairments in memory functions. In addition, previous studies found that early life stress in particular is associated with lower hippocampal volume (68). As cPTSD is often associated with trauma in early childhood (1, 55, 61), cognitive impairments in learning and memory in cPTSD may be the result of reduced hippocampal volume due to trauma experienced during childhood. Regarding this assumption, a previous study already highlighted the need to empirically compare cognitive profiles of cPTSD patients with and without childhood trauma for future research (69). These more severe memory impairments in patients with cPTSD are highly relevant, as previous studies were able to demonstrate that learning and memory deficits in patients with PTSD are associated with problems in everyday life and therapy (70). For example, memory deficits in PTSD were a predictor for problems in social and occupational functioning (65). Moreover, pre-treatment learning and memory performance can predict treatment outcomes in PTSD, which was particularly important for verbal memory (71–73). Haaland et al. (2016) suggest interventions to improve learning and memory before therapy (72).

Concerning the cognitive domain of attention, we found significant group differences in selective attention, with slower reaction times in the cPTSD group. This is in line with previous research that revealed greater impairments of attention in cPTSD compared to ncPTSD with an association with symptom severity (21). Based on previous cognitive models of PTSD, these attentional deficits may be the consequence of a “shift” in information processing capacities toward the search and recognition of potentially threat-related stimuli, causing a focus on the traumatic experience and a reinforcement of PTSD symptoms (15, 74, 75). The heightened attentional focus on threat-related stimuli is assumed to be established at the expense of attention on other stimuli and the expense of other cognitive domains (11, 15). Thus, greater impaired attention in cPTSD may be associated with greater impairments in other domains of cognition, such as memory. Moreover, addressing the attentional bias toward threat stimuli, e.g. by applying attention control training, was shown to reduce PTSD symptoms (76).

Contrary to our expectations and prior studies, no significant group differences in cognitive functioning were observed between patients with cPTSD and ncPTSD with regard to executive functioning and the attentional subdomains of divided attention and processing speed. In particular, complex trauma, often involving repeated interpersonal threats in the early stages of life, was previously associated with impaired executive functioning and processing speed (77). A study investigating patients with a history of parental abuse revealed that the patients with cPTSD performed significantly worse in working memory, a subdomain of executive functioning, than those with ncPTSD (21). However, a recent study investigating a sample of veterans also showed more deficits in executive functioning in cPTSD compared to ncPTSD (22). Previous research demonstrated that the duration and type of trauma may influence the manifestation of posttraumatic symptoms, impacting cognitive functioning as well (11, 15). Considering these findings, the type of trauma might have differential effects on cognitive functioning in cPTSD patients, which, however, has not yet been explicitly investigated. To gain a deeper understanding of cognitive functioning in patients with cPTSD, future research should therefore consider the type of trauma as a potentially relevant variable.

### 4.3 Factors influencing cognitive impairment

We identified a higher age to be significantly associated with impairments in visual memory and selective attention of trauma patients. Previous research in trauma patients has already identified the variable age to be negatively associated with cognitive performance (9, 78). This may be attributed to age in general being accompanied by cognitive decline (79) or to the duration of PTSD symptoms (9). In a longitudinal study on cognitive performance in older patients with PTSD, Yehuda et al. (2006) found that aging, trauma exposure, and PTSD symptom severity may have differential effects on memory performance (78).

Analyzing the variables in one model, the type of PTSD (cPTSD vs. ncPTSD) was identified to be a predictor of cognitive performance in visual memory and selective attention. This outcome is not surprising, given that we selected variables for regression analysis based on the presence of significant group differences. However, the lack of significant influence from comorbidities in this model suggests that it is not the individual variables but rather the interplay of different factors being associated with cognitive impairment in patients suffering from posttraumatic stress. Additionally, psychotropic medication was found to be associated with better performance in selective attention in our model. Previous research has shown mixed results regarding the impact of psychotropic medication on cognition. While antidepressants may positively influence certain cognitive domains, such as psychomotor speed and delayed recall (80), other studies found a negative effect of psychotropic medication on cognitive functioning. For instance, psychotropic medication has been associated with lower overall cognitive function and working memory (81), and benzodiazepine use has been linked to cognitive deficits (82). To get a better understanding of these effects, future research on cognition in PTSD should further consider specific types of medication, including dosage and duration of intake.

## 5 Limitations

An important limitation of the study is that its statistical power is sufficient to detect large effect sizes. However, based on previous research, medium effect sizes may also be hypothesized in comparing cognitive functioning in trauma patients (10). Another limitation is that the study population was homogenous in terms of gender, nationality, and type of trauma limiting the generalizability of the study results. The participants were mainly female and of German nationality, with various other forms of trauma, such as war-related trauma, remaining underrepresented. Furthermore, a limitation can be considered in the study’s cross-sectional design limiting the generalizability of our findings regarding possible longitudinal variable changes. Moreover, this research shares limitations with other studies on cognitive functioning in trauma patients concerning the possibility of earlier alcohol, drug, or medication abuse that was not reported by the participants and may have affected cognitive performance (65, 67, 83). Moreover, studies indicate that PTSD treatment might affect cognition. For example psychotherapy such as cognitive behavioral therapy has been associated with improvement in memory function (71). The majority of our participants had received psychotherapy (cPTSD 94%, ncPTSD 83%), resulting in a relatively homogeneous group in terms of treatment exposure. It would be interesting in future studies to compare groups with and without prior psychotherapy to explore differences in cognitive outcomes. Another limitation of the present study is that the regression analysis only considered a selection of possible variables. Our choice was made based on existing literature, but other factors may also influence cognitive impairment in trauma patients.

## 6 Conclusion

The finding of differences in cognitive functioning between cPTSD and ncPTSD diversifies our understanding of the multifaceted symptomatology that may result from trauma. Based on the result that cPTSD patients displayed a higher symptom burden and more pronounced cognitive deficits, especially in visual memory and selective attention, more intense and comprehensive interventions may prove to be beneficial for treatment. As cognitive functioning plays an important role in processing traumatic memories and engaging in therapeutic processes, cognitive training should be implemented as part of a comprehensive treatment plan.

## Data Availability

The datasets presented in this study can be found in heiDATA, https://doi.org/10.11588/data/B3HNSQ.
